# Choroidal neovascularization secondary to angioid streaks in a patient with pseudoxanthoma elasticum: case report

**DOI:** 10.22336/rjo.2024.85

**Published:** 2024

**Authors:** David-Ionuț Beuran, Camelia Constantin, Răzvan Constantin Șerban, Lucian George Eftimie, Cătălin Cornăcel

**Affiliations:** 1 Department of Ophthalmology, „Dr. Carol Davila” Central Military University Emergency Hospital, Bucharest, Romania; 2 Department of Dermatology, „Dr. Carol Davila” Central Military University Emergency Hospital, Bucharest, Romania; 3 Department of Pathology, „Dr. Carol Davila” Central Military University Emergency Hospital, Bucharest, Romania; 4 Discipline of Anatomy and Biomechanics, National University of Physical Education and Sports, Bucharest, Romania

**Keywords:** angioid streaks, choroidal neovascularization, pseudoxanthoma elasticum, PXE = Pseudoxanthoma elasticum, PPi = Inorganic plasma pyrophosphate, AS = Angioid streaks, CNV = Choroidal neovascularization, FA = Fluorescein angiography, FAF = Fundus autofluorescence, SD-OCT = Spectral-domain optical coherence tomography, LE = Left eye, RE = Right eye, IOP = Intraocular pressure, NCT = Non-contact tonometer, BE = Both eyes, RPE = Retinal pigment epithelium, ABC = ATP-binding cassette, ATP = adenosine triphosphate, Anti-VEGF = Anti-vascular endothelial growth factors, CRT = Central retinal thickness

## Abstract

**Objective:**

Present the diagnosis and therapeutic approach in a patient with pseudoxanthoma elasticum and ocular involvement.

**Case report:**

A 46-year-old patient presented for progressive loss of vision and metamorphopsias in the left eye. The ophthalmological examination showed angioid streaks and secondary choroidal neovascularization. The dermatologist performed an incisional biopsy of a skin area in the right axilla that showed white-yellow, discrete streaks. Histopathological examination confirmed the diagnosis of pseudoxanthoma elasticum. It was decided to start intravitreal injections with aflibercept. Three injections were made in the left eye with subsequent visual acuity improvement.

**Discussion:**

Angioid streaks are a rare retinal condition, and pseudoxanthoma elasticum is the most common association. Other systemic conditions are Ehlers-Danlos syndrome, Paget’s disease, and hemoglobinopathies. Definite diagnosis requires skin lesions or calcified elastic fibers on pathological examination and angioid streaks of the retina. Choroidal neovascularization is a frequent complication and leads to vision loss. Intravitreal inhibitors of vascular endothelial growth factor are currently the first line of treatment.

**Conclusions:**

Pseudoxanthoma elasticum has numerous systemic manifestations and requires a multidisciplinary team to be monitored and treated.

## Introduction

### Background

Pseudoxanthoma elasticum (PXE) is a hereditary disease characterized by calcification and fragmentation of connective tissue [1]. It is distinguished by lower levels of inorganic plasma pyrophosphate (PPi), a calcification inhibitor [2]. The most affected structures are the skin, retina (Bruch’s membrane), blood vessels, and gastrointestinal tract [3].

Angioid streaks (AS) are a rare retinal condition and consist of irregular, linear, crack-like dehiscences of a calcified and brittle Bruch’s membrane [4,5]. The condition is often bilateral [5].

### Epidemiology and associations

A population-based longitudinal cohort study involving 126 million individuals was conducted in Japan. During the 10-year study period, 6598 AS and 1020 cases of PXE were identified. The incidence was 0.52 per 100,000 person-years for AS and 0.08 per 100,000 for PXE. The overlap of AS and PXE was observed in 363 patients [6].

A retrospective cross-sectional study included 1852 cases of AS. The association between AS and systemic conditions was the following: Pseudoxanthoma elasticum (PXE) in 228 patients (12.3%), Ehlers-Danlos syndrome in 18 patients (1%), Paget’s disease in six patients (0.3%), hemoglobinopathies 30 patients (1.6%), and idiopathic 1573 patients (84,9%) [7].

### Clinical features

In PXE, skin manifestations consist of discrete papules with a yellowish hue located mainly on the lateral neck, axillae, and antecubital and popliteal fossae. These lesions gradually coalesce into big plaques [8].

Fundus examination reveals the following: peau d’orange, AS, peripheral comet and comet tail lesions, choroidal neovascularization, pattern dystrophy-like changes, chorioretinal atrophy, and optic nerve head drusen [9]. Peau d’orange is the earliest change characterized by a mottled aspect of the retinal midperiphery. It represents a transition between calcified and non-calcified Bruch’s membrane and is most commonly located temporarily. AS are reddish or brownish irregular lines originating at the optic nerve, radiating to the periphery. A peripapillary ring of atrophy can be observed. AS may lead to choroidal neovascularization (CNV) with consecutive complications. Comet lesions are small, round, peripheral chorioretinal atrophies. Comet tail lesions have a tail pointing toward the optic nerve. The findings are generally bilateral [9,10].

Progressive mineralization of the elastic media and intima in mid-sized arteries causes vascular involvement. The main clinical manifestations are loss of peripheral pulses, intermittent claudication, renovascular hypertension, acute upper gastrointestinal hemorrhage, intestinal angina, and coronary artery disease [8].

### Paraclinical features

Although the diagnosis of AS is primarily clinic, other changes such as CNV and optic nerve drusen are better visualized using fluorescein angiography (FA), fundus autofluorescence (FAF), and spectral domain optical coherence tomography (SD-OCT) [11].

## Case report

### Ophthalmological examination

A 46-year-old Caucasian female presented for progressive loss of vision and metamorphopsias in the left eye (LE).

From the history, it was noted that the patient was diagnosed with angioid streaks 20 years before. The patient explained that she was treated with laser at the time of diagnosis, but she could not present documents.

**Fig. 1:**
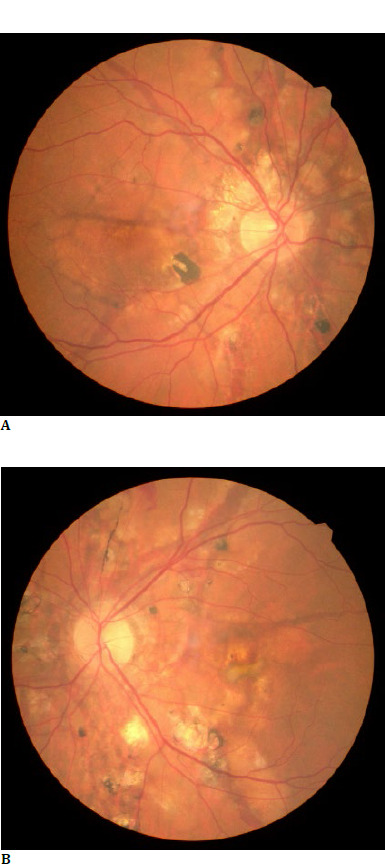
**A**. Fundus photo of the RE; **B**. Fundus photo of the LE

When examined, best-corrected visual acuity (BCVA) was 1 in the right eye (RE) and 0,3 in the LE after correction of moderate myopia with the following refraction for the RE: -3,75/-0,25/156° and for the LE: -5,25/-0,25/32°. The intraocular pressure (IOP) was 20 mm Hg for the RE and 21 mm Hg for the LE, measured with a non-contact tonometer (NCT). No pathological changes were revealed at the anterior pole examination. Posterior pole examination revealed bilateral irregular brown lines radiating from the optic disc. Furthermore, chorioretinal atrophy and hyperpigmentation areas were described for both eyes (BE) (**Fig. 1 A, B**). For the LE, peripheral retinal hemorrhage and a macular green-brown lesion were observed (**Fig. 1 B**).

**Fig. 2:**
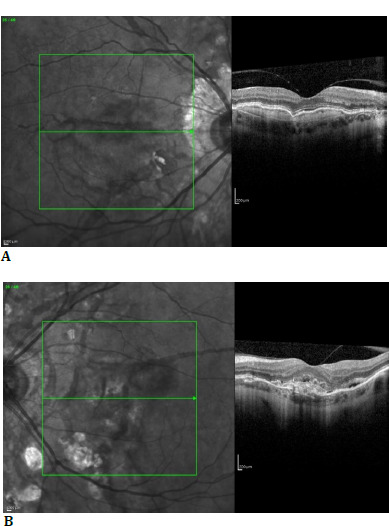
**A**. Macula OCT of the RE; **B**. Macula OCT of the LE

Macula OCT revealed for BE a hyperreflective line in the vitreous cavity attached to the fovea and an interrupted Bruch’s membrane corresponding to angioid streaks (**Fig. 2 A, B**). RE macula OCT revealed a slightly hyperreflective, homogeneous structure between the retinal pigment epithelium (RPE) layer and Brunch’s membrane (**Fig. 2 B**). Furthermore, similar changes were observed for the LE but inhomogeneous, with EPR interruption and subretinal fluid (**Fig. 2 B**).

After ophthalmological examination, the following diagnoses were made: BE - moderate myopia, presbyopia, angioid streaks, choroidal neovascularization; RE - type I inactive, LE - type II active.

### Dermatology, cardiology, and histopathology examination

The dermatological examination revealed white-yellow, discrete streaks disseminated at the bilateral axillary level. An incisional biopsy of a lesion was performed for histological examination.

**Fig. 3:**
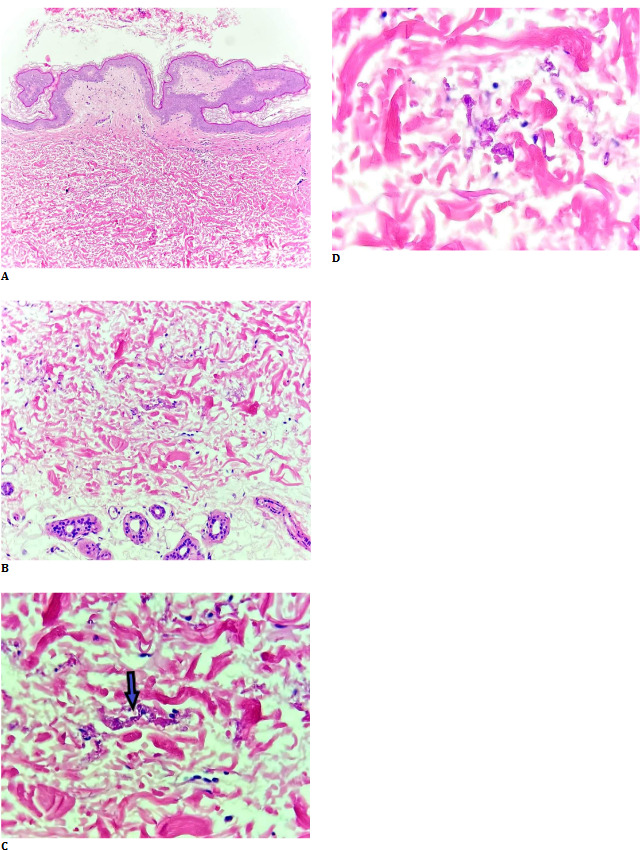
**A-D**. Hematoxylin and eosin stained sections of the biopsy. Elastic fibers are short and fragmented, with abnormal basophilic fibers (arrow)

Histopathology described the skin biopsy fragment. The affected elastic fibers exhibited basophilia and irregular morphology, presenting as widely scattered granular material interspersed among normal collagen fibers, occasionally resembling the distinctive shape of a bishop’s crook. Abnormal fibers displayed a bright pink hue with disrupted architecture, characterized by the loss of their typical interwoven pattern, although they might have exhibited faint basophilia due to calcium deposition. Vascular involvement was marked by fragmentation of the internal and external elastic laminae and intimal thickening, leading to vessel wall weakening and an increased propensity for rupture and aneurysm formation. The reticular dermis’s elastic fibers were short and fragmented, with abnormal basophilic fibers visible in hematoxylin and eosin (H & E) stained sections (**Fig. 3 A-D**).

Given the rarity of this pathology, the case was referred for consultation to Phillip McKee, MD, FRCPath, a world-renowned dermatopathologist, for diagnostic confirmation.

For differential diagnosis, opinions were sought from other dermatopathologists through the Dermatopathology working group “McKee Derm”. They concurred with the diagnosis of PXE.

The presence of angioid streaks larger than an optic disc diameter, skin findings, and histopathological changes confirmed the diagnosis of PXE of mild severity [3].

Cardiology examination concluded the following diagnoses: grade II hypertension, high added risk, and extrasystolic arrhythmia Lown class II. Furthermore, computed tomographic angiography isolated calcareous plaques at the level of the abdominal aorta.

### Ophthalmological treatment

**Fig. 4:**
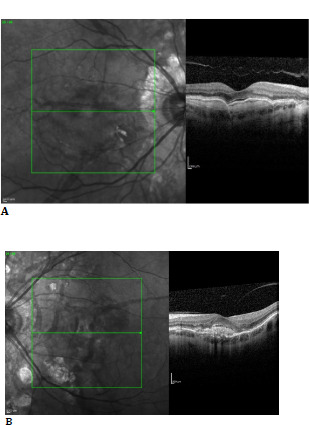
**A**. Macula OCT of the RE 3 months after the first examination; **B**. Macula OCT of the LE after three intravitreal injections with aflibercept

It was decided to start intravitreal injections with aflibercept. Three doses of 0,05 ml were injected at 4 mm posterior to the limbus in the LE. The BCVA maintained 1 in the RE and improved to 0,9 in the LE. OCT was stationary in the RE (**Fig. 4 A**). In the LE, subretinal fluid was reduced in dimensions (**Fig. 4 B**).

## Discussion

### Genetics and mechanisms

Different genetic variants of PXE were described: ABCC6, ENPP1, and GGCX [8]. The ABCC6 gene is translated into a 165 kDa protein, ABCC6, a member of the C family of ATP-binding cassette (ABC) proteins. ABCC6 works as an efflux transporter localized primarily in hepatocytes [12]. This protein promotes the efflux of adenosine triphosphate (ATP), and the low activity of this function leads to low levels of PPi, a critical anti-mineralization factor [13].

### Differential diagnosis

It can be made for dermatological lesions, ocular manifestations, and vascular involvement. After diagnoses of angioid streaks, it is essential to determine the systemic association, which occurs in up to 50% of the patients [14]. The leading systemic associations are PXE, Ehlers-Danlos syndrome, Paget’s disease, hemoglobinopathies (b-thalassemia), and sickle cell disorder [7]. Furthermore, in the case of PXE, genetics helps determine clinicopathological features and predominantly affected organs. ENPP1 is associated with generalized arterial calcification of infancy in overlap with PXE. GGCX involves vitamin K-dependent coagulation defects [8].

### Treatment and complications

Choroidal neovascularization is the main ocular complication, and anti-vascular endothelial growth factor (anti-VEGF) agents are the first line of treatment at present. In the past, argon laser photocoagulation and photodynamic therapy were used with unsatisfactory results. Ranibizumab, aflibercept, and off-label bevacizumab are the three main anti-VEGF agents used [15].

Aflibercept was administered for active neovascularization in 23 eyes of 20 patients. A patient received one dose initially and a *pro-re-nata* regimen afterward for 48 weeks. The central retinal thickness (CRT) was reduced (p=0,03), and 18 eyes (81,8%) featured stability within 15 letters [16].

Another 12-month prospective clinical trial included 15 PXE patients with active CNV. They received one initial injection and a *pro-re-nata* regimen with monthly examinations. BCVA improved (p=0,083), and CRT decreased (p=0,004) [17].

Fourteen eyes of 13 patients previously treated with intravitreal ranibizumab for refractory or recurrent choroidal neovascularization were switched to intravitreal aflibercept. After 12 months of follow-up, CRT decreased (p=0,008), and 71% of patients did not have intraretinal/subretinal fluid [18].

### Prognosis

Classic CNV has a worse prognosis because it may develop a fibrotic scar, with consequent worsening of visual acuity [19]. Furthermore, a study reported a relatively poor long-term visual prognosis in this patient type. Thirty-three eyes of 23 patients were included. BCVA decreased during treatment with anti-VEGF agents (mean follow-up duration: 109 ± 42 months) [20].

## Conclusions

Pseudoxanthoma elasticum is a disease with numerous systemic manifestations. It requires a multidisciplinary team to monitor and treat. Furthermore, the ophthalmological examination can be the first step in diagnosing it.
